# A novel potential target of IL‐35‐regulated JAK/STAT signaling pathway in lupus nephritis

**DOI:** 10.1002/ctm2.309

**Published:** 2021-02-01

**Authors:** Zhe Cai, Song Zhang, Ping Wu, Qi Ren, Ping Wei, Ming Hong, Yu Feng, Chun Kwok Wong, Hong Tang, Huasong Zeng

**Affiliations:** ^1^ The Joint Center for Infection and Immunity, Guangzhou Institute of Pediatrics Guangzhou Women and Children's Medical Center, Guangzhou Meidcal University, Guangzhou, China Institute Pasteur of Shanghai Chinese Academy of Science Shanghai China; ^2^ Department of Allergy Immunology and Rheumatology Guangzhou Women and Children's Medical Center, Guangzhou Meidcal University Guangzhou China; ^3^ Department of Chemical Pathology Prince of Wales Hospital The Chinese University of Hong Kong Hong Kong China; ^4^ Institute of Chinese Medicine and State Key Laboratory of Research on Bioactivities and Clinical Applications of Medicinal Plants The Chinese University of Hong Kong Hong Kong China; ^5^ Jinan University Guangzhou China; ^6^ Institute of Advanced Diagnostic and Clinical Medicine, Zhongshan People's Hospital Guangzhou University & Zhongshan People's Hospital Joint Biomedical Institute 2 Sunwen East Road Zhongshan China; ^7^ Department of Traumatology Ningxia Hui Autonomous Region General Hospital of Ningxia Medical University Yinchuan China; ^8^ Institute Pasteur of Shanghai Chinese Academy of Science Shanghai China

**Keywords:** IL‐35, JAK/STAT signaling pathway, JSLE‐LN, LAIR1, mesangial calls

## Abstract

**Background:**

In this study, we have investigated the potential regulatory mechanisms of IL‐35 to relieve lupus nephritis (LN) through regulating Janus kinase (JAK)/signal transducers and activators of transcription (STAT) signaling pathway in mesangial cells.

**Results:**

Among 105 significant differentially expressed proteins (DEPs) between juvenile systemic lupus erythematosus (JSLE) patients with LN and healthy controls, LAIR1, PDGFRβ, VTN, EPHB4, and EPHA4 were downregulated in JSLE‐LN. They consist of an interactive network with PTPN11 and FN1, which involved in IL‐35‐related JAK/STAT signaling pathway. Besides, urinary LAIR1 was significantly correlated with JSLE‐LN clinical parameters such as SLEDAI‐2K, %CD19+ B, and %CD3+ T cells. Through bioinformatics analysis of co‐immunoprecipitation with mass spectrometry results, including GO, KEGG, and STRING, five genes interacted with Lair1 were upregulated by IL‐35, but only Myh10 was downregulated. Therefore, we presumed an interactive network among these DEPs, JAK/STAT, and IL‐35. Moreover, the downregulated phosphorylated (p)‐STAT3, p‐p38 MAPK, and p‐ERK, and the upregulated p‐JAK2/p‐STAT1/4 in IL‐35 overexpressed mesangial cells, and RNA‐sequencing results validated the potential regulatory mechanisms of IL‐35 in alleviating JSLE‐LN disease. Moreover, the relieved histopathological features of nephritis including urine protein and leukocyte scores, a decreased %CD90^+^αSMA^+^ mesangial cells and pro‐inflammatory cytokines, the inactivated JAK/STAT signals and the significant upregulated Tregs in spleen, thymus and peripheral blood were validated in Tregs and IL‐35 overexpression plasmid‐treated lupus mice.

**Conclusions:**

Our study provided a reference proteomic map of urinary biomarkers for JSLE‐LN and elucidated evidence that IL‐35 may regulate the interactive network of LAIR1‐PTPN11‐JAK‐STAT‐FN1 to affect JAK/STAT and MAPK signaling pathways to alleviate inflammation in JSLE‐LN. This finding may provide a further prospective mechanism for JSLE‐LN clinical treatment.

## Graphical abstract

A reference proteomic map of urinary biomarkers contributes to the diagnosis of JSLE‐LN.

IL‐35 may interact with LAIR1‐PTPN11‐JAK‐STAT‐FN1 to affect JAK/STAT and MAPK signaling pathways to alleviate inflammation in vivo and in vitro.

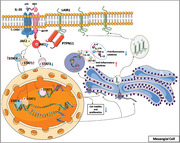

**Abbreviations**
ACN: acetonitrile
ANA: antinuclear antibody
COG: clusters of orthologous groups
Co‐IP: co‐immunoprecipitation
CTX: cyclophosphamide
DEGs: differentially expressed genes
DEPs: differentially expressed proteins
dsDNA: double stranded DNA
ELISA: enzyme‐linked immunosorbent assay
EPHA4: ephrin type‐A receptor 4
EPHB4: ephrin type‐B receptor 4
FN1: fibronectin 1
GO: gene ontology
IPA: ingenuity pathway analysis
JAK: Janus kinase
JSLE: juvenile systemic lupus erythematosus
KEGG: Kyoto Encyclopedia of Genes and Genomes
LAIR1: leukocyte‐associated immunoglobulin like receptor 1
LC: liquid chromatography
LN: lupus nephritis
MAPK: mitogen‐activated protein kinase
MS: mass spectrometry
MTX: methotrexate
NC: normal control
PBMC: peripheral blood mononuclear cell
PBS: phosphate buffer saline
PDGFRβ: platelet‐derived growth factor receptor beta;
PPI: protein‐protein interaction
PTPN11: protein tyrosine phosphatase non‐receptor type 11
RBC: red blood cell
SH2: Src homology 2
SLEDAI: SLE disease activity index
STAT: signal transducer and activator of transcription
STRING: search tool for the retrieval of interacting genes
VTN: vitronectin

## 1 BACKGROUND

Systemic lupus erythematosus (SLE) is characterized by a chronic autoimmune disease with massive inflammation that often results in multiple organs damages, especially renal damages in lupus nephritis (LN).^1^, ^2^, ^3^ The precise pathogenic mechanism of SLE is largely unknown. Despite tremendous basic and clinical research progress regarding treatment,^1^ various cytokines and chemokines are emerged as the potential biomarkers of disease activity.^4^ However, due to the complexity of SLE, the clinical application of these potential biomarkers in assessing the activity of disease is not well established, especially through assessing the noninvasive and easily collected urine samples to diagnose LN activity in SLE. In an attempt to reach a greater understanding of SLE pathogenesis, based on the advantage of proteomic and RNA‐sequencing (RNA‐seq) technologies with bioinformatics analysis, which could explore the underlying mechanisms of disease, the potential biomarkers in urine samples were investigated to differentiate juvenile SLE (JSLE) patients with inactive and active LN from healthy donors. For example, leukocyte‐associated immunoglobulin (Ig)‐like receptor‐1 (LAIR1) is one of the significantly downregulated differential expression proteins (DEPs) in JSLE patients.^5^, ^6^ LAIR1 performs a potential inhibitory effect on lymphocytes by its two cytoplasmic tyrosine‐based inhibitory motifs binding to the Src homology 2 (SH2) domain of SH2‐containing protein tyrosine phosphatase (PTP)‐2 (SHP2, PTPN11), and leading to the dephosphorylation of subsequent kinases.^7^, ^8^, ^9^

Recently, a study demonstrated that the activity of PTPN11 on peripheral blood mononuclear cells (PBMCs) was increased in both lupus‐prone MRL/*lpr* mice and SLE patients.^10^ While, inhibiting the activity of PTPN11 could alleviate splenomegaly, suppress glomerulonephritis, and increase the life span of lupus mice.^10^ It indicated that PTPN11 should be a critical regulator involved in the pathogenesis and development of SLE. Therefore, targeting inhibition of the activity of PTPN11 could be a potent treatment for SLE patients with LN. Although several studies indicated that LAIR1 could transduct the inhibitory signals through recruiting PTPN11 to inhibit the JAK/STAT signaling pathway in leukocytes,^11^, ^12^ the LAIR1 and PTPN11‐regulated signaling pathway and the inhibitory function of LAIR1 in mesangial cells of JSLE‐LN are still not well investigated. Interestingly, in our previous investigation, we have mentioned the STAT1‐STAT4 could constitute to be an unique heterodimer and bind to the distinct promoter regions of genes IL‐12A (p35) and Epstein‐Barr virus induced (Ebi)‐3, which were the two subunits of interleukin (IL)‐35. The process of STAT1‐STAT4 regulating the expression of p35 and Ebi3 was closely related to the anti‐inflammatory mechanism of IL‐35.^2^ Thereby, we may presume if there is a regulatory relationship among IL‐35, LAIR1, and PTPN11 in JSLE‐LN disease, they should form an interactive network via JAK/STAT signaling pathway. However, this assumption should be verified further.

IL‐35, the newest member of IL‐12 family, is distinct from its siblings like IL‐23 and IL‐27. It has an anti‐inflammatory effect on activating the regulatory T cells (Tregs) and directly suppressing effector T cells proliferation with or without regulatory cytokines such as IL‐10 or TGF‐β in vitro.^13^ Similar to IL‐27 transducing signals by binding to its receptor, IL‐35 subunits p35 and EBI3 would bind to chains IL‐12Rβ2 and gp130, respectively. In this perspective, the gp130 and IL‐12Rβ2 chains would constitute to be a heterodimer (IL‐12Rβ2:gp130), which in fact was the IL‐35 receptor (IL‐35R).^14^ Meanwhile, through the interaction of IL‐35R with JAK1/2 and STAT1/3/4, the extracellular signal could be converted to regulate the downstream gene expression and intracellular factors.^2^ There are little studies on the immunoregulatory signaling pathway of IL‐35 in the modulation of JSLE‐LN. We have proved the direct and indirect anti‐inflammatory effect of IL‐35 on cytokine‐mediated inflammation of SLE.^2^, ^3^ In order to further elucidate the potential target of Treg cells secreted IL‐35, which can regulate the inflammation of SLE,^15^ we have investigated the possible relationships among LAIR1, IL‐35, and JAK/STAT signaling pathways in vitro and in vivo. In this study, we can infer that IL‐35 (EBI3‐p35) and IL‐35R (IL‐12Rβ2‐gp130) may interact with JAK‐STAT‐PTPN11‐LAIR1 to affect JAK/STAT signaling pathway in alleviating inflammation of JSLE‐LN. These results could form a biochemical basis for the application of IL‐35 to treat JSLE‐LN disease in future and for the exploration of its potential targets in clinical diagnosis.

## 2 MATERIALS AND METHODS

### 2.1 Patients, samples collection, and clinical manifestations

The clean morning spot urine samples were collected from total 19 JSLE‐LN patients (nine inactive JSLE‐LN and 10 active JSLE‐LN) and nine healthy controls. All subjects were enrolled in the Guangzhou Women and Children's Medical Center according to the disease activity as reflected by the SLE Disease Activity Index (SLEDAI)‐2K, serum levels of anti‐nuclear antibody (ANA) and anti‐double stranded DNA (anti‐dsDNA) antibody. The written informed consents were obtained from all subjects according to the Declaration of Helsinki. The protocol was approved by the Ethics Committee of Guangzhou Women and Children's Medical Center. Patients’ information regarding demographic characteristics, clinical parameters, serological profiles, and medications were retrieved from medical records. Therefore, the association between the DEPs and clinical parameters of JSLE‐LN patients were determined.

### 2.2 Sample preparation for liquid chromatography‐tandem mass spectrometry measurement

For liquid chromatography (LC)‐mass spectrometry (MS/MS) analysis, each isolated protein sample from midstream urine collected from each subject was reduced and digested for fractionation. Each fraction was resuspended and separated in an Ultimate 3000 nano‐LC system (Dionex). Then, eluted peptides were injected directly to Q Exactive Hybrid Quadrupole‐Orbitrap MS system (Thermo Fisher Scientific). Through a nanoelectrospray ion source (Proxeon Biosystems), MS spectra were acquired. Using high energy collision dissociation operating mode, the peptides were selected for MS/MS.

### 2.3 Proteomics analysis

MaxQuant software (version 1.0.13.13) was used to extract the intensities and unique peptide counts for protein identification and quantification from LC‐MS/MS raw data. All proteins (adjusted p‐value < 0.05) were assessed in the Ingenuity Pathway Analysis software (IPA, www.ingenuity.com) for proteomics analysis based on published reports and databases such as gene ontology (GO) database (http://www.ebi.ac.uk/QuickGO/), Kyoto Encyclopedia of Genes and Genomes (KEGG, https://www.genome.jp/kegg/kegg2.html), Uniport, TrEMBL and the search tool for the retrieval of interacting genes (STRING) online database (https://string‐db.org/cgi/input.pl).

### 2.4 Co‐immunoprecipitation assay and immunoblot analysis

For the co‐immunoprecipitation (Co‐IP) assay, three repeats of five different treated mesangial cells were digested and collected to extract proteins. The immunoprecipitates were subjected to MS/MS examination for identifying Lair1 interactive proteins. In brief, the DEPs were analyzed in a cloud workshop platform (Shanghai Majorbio Bio‐pharm Technology Co., Ltd.) and the Venny 2.1 online (https://bioinfogp.cnb.csic.es/tools/venny/index.html).

### 2.5 Plasmid constructs and transfection

The DNA fragments of mouse IL‐35 (IL‐12A‐Ebi3) and short hairpin RNA (shRNA), which was designed to knockdown mouse Ebi3 expression, were cloned into the PGMLV‐6395 eukaryotic expression plasmid. This PGMLV‐6395 plasmid was used as the IL‐35 null vector (IL‐35n). These reconstructed plasmids were confirmed by DNA sequencing. The plasmids were transfected into 293T cells according to a standard protocol to verify their bioactivity by Genomeditech company (Genomeditech, Shanghai, Co., Ltd.). Then the secreted IL‐35 from supernatant was verified by enzyme‐linked immunosorbent assay (ELISA) with mouse IL‐35 heterodimer ELISA Kit (BioLegend, San Diego, CA, USA). Therefore, the functional vectors of IL‐35 overexpression plasmid (IL‐35p), IL‐35n, and shRNA plasmid could be transfected into mesangial cells for further investigation.

### 2.6 Isolation of glomerulus and incubation of mesangial cells

The primary mesangial cells were isolated from the kidney of sacrificed female Balb/c mice (n = 2) in age of 4 weeks by following a standard protocol.^16^, ^17^, ^18^ Freshly isolated kidneys were washed and digested into glomerulus pellet. After removing tubulars by filtrating and rinsing until the glomerulus reached 99% purity, the purified glomerulus were transferred to flask for mesangial cell culture with Dulbecco's modified Eagle's medium (DMEM)/F12 medium (Life Technologies, Grand Island, NY) supplemented with 10% fetal bovine serum. After 30 days of growth, the pure mesangial cells could be confirmed by morphology and flow cytometry (Navios, Beckman Coulter Inc., Brea, CA, USA) detection with specific markers CD90 and αSMA (BioLegend).

### 2.7 The cell viability of mesangial cells

Mouse recombinant IL‐35 (1 μg) was dissolved in 100 μL DMEM/F12 medium and then diluted into various concentrations (0 to 200 ng/mL). The viability of CD90^+^αSMA^+^ mesangial cells treated with different concentrations of IL‐35 for 24 and 48 hours was assayed by Cell Proliferation Kit II (Sigma‐Aldrich, Merck Millipore, Billerica, MA, USA).

### 2.8 Quantitative real‐time polymerase chain reaction (RT‐qPCR) detection of mesangial cells with different treatments

There were five ways to treat mesangial cells including the shRNA of IL‐35 transfection (shRNA), shRNA with 12.5 ng/mL mouse recombinant IL‐35 treatment (shRNA+12.5 ng/mL IL‐35), IL‐35 overexpression plasmid transfection (IL‐35 plasmid), 12.5 ng/mL mouse recombinant IL‐35 treatment (12.5 ng/mL IL‐35), and normal control (NC). After 8 hours treatment, the mesangial cells were collected for RNA isolation using the RNeasy Mini Kit (Qiagen GmbH, Hilden, Germany) according to the manufacturer's protocol. The isolated total RNA was qualified by using NanoDrop2000 and Agilent 2100 Bioanalyzer (Agilent Technologies) to exclude genomic DNA contamination and poorly degraded RNA. All eligible RNA samples were processed for RT‐qPCR using One Step SYBR PrimeScript RT‐PCR kit II (Takara Bio Inc., Otsu, Shiga, Japan) and Applied Biosystems 7900HT Fast Real‐Time PCR System (Applied Biosystems Inc., Foster City, CA, USA). The relative mRNA expression was measured using the comparative CT (Threshold Cycle) and calculated by comparing to the expression of GAPDH using a formula [2^–ΔCt (Ct^_target gene_^–Ct^_GAPDH_^)^].

### 2.9 RNA‐seq of mesangial cells with different treatments

A total of 50 RNA samples from mesangial cells with above treatments together with five different time points (4, 8, 24, 48, and 96 hours) were performed for RNA‐sEquation (Shanghai Majorbio). Two repeats in each time points. The cDNA library was prepared using Illumina TruseqTM RNA sample preparation kit. The final cDNA library was created for paired end sequencing using Illumina1Hiseq‐2000 platform.

### 2.10 RNA‐seq analysis

Original RNA‐seq and quality control data were processed for pre‐alignment followed with the statistics analysis. After quality control assessment, the clean data were aligned to the reference mouse genome and gene model downloaded from Ensembl database (https://asia.ensembl.org/Mus_musculus/Info/Index) using TopHat2 program (version 2.1.1, http;//tophat.cbcb.umd.edu/) with default parameters. Cufflink program (version 2.0.2, http://cole‐trapnelllab.github.io/cufflinks) was used to determine differentially expressed genes (DEGs) and transcripts. A difference of at‐least two fold in the transcripts/genes expression between different treatments and time points was considered for further analysis. Furthermore, using DESeq2 program to analyze DEGs and compare the results of Cufflink analysis, we took the intersection of them for gene set analysis and downstream pathway annotation.

### 2.11 Bioinformatics analysis

The clusters of orthologous groups (COG), GO, KEGG annotation combined with Venn, heat map, and STRING analysis were applied to explore the potential protein–protein interaction (PPI) network among IL‐35, JAK/STAT, and Lair1. The DEGs were used to generate heat map that cluster genes with similar expression patterns together. These genes involved in the presumed signaling pathway were classified as significantly elevated if their p‐value < 0.05 and fold‐change ≥ 2 or ≤ 0.5. The data from each group were imported into Heml software (version 1.0) for clustering analysis and heat map generation. For clustering, genes were clustered in an unsupervised manner based on Euclidean distance.

### 2.12 Lupus mice and injection protocol

Totally 30 4‐week‐old female MRL/MpJ‐Faslpr/2J (MRL/lpr) lupus mice and five sex‐ and age‐matched Balb/c mice were bred and kept in a conventional animal facility under specific pathogen‐free conditions in the Laboratory Animal Services Center, The Chinese University of Hong Kong (LASC, CUHK). All experiments involving live animals were carried out strictly according to the principles outlined in the Animal Experimentation Ethics Committee Guide for the Care and Use of Laboratory Animals, as approved by the Animal Experimentation Ethics Committee of CUHK. MRL/lpr lupus mice (n = 5/group) were injected intravenously with a single dose of 100 μL/mouse purified IL‐35p and IL‐35n, as well as 10^6^/mouse purified Tregs. The mice (n = 5/group) injected intraperitoneally (once per week) with 2 mg/kg methotrexate (MTX) and 90 mg/kg cyclophosphamide (CTX), respectively, were the positive control. The phosphate buffer saline (PBS)‐treated lupus mice as the negative control group was injected intraperitoneally once per week.

### 2.13 Monitoring disease activity

Urine collected from each group (n = 5) of MRL/lpr mice was analyzed for protein, leukocyte, and blood using the Mission Urinalysis Reagent Strip (ACON Biotech, Co., Ltd., Hangzhou, Zhejiang, China). According to a modified macroscopic scoring system of urinalysis for JSLE‐LN (Table S1), the proteinuria, leukocyturia, and urine blood were demonstrated.^5^, ^19^, ^20^ The body weight of each mouse was measured the same as urine test did once a week for 12 weeks. These detection periods covered a life‐span of lupus mouse from juvenilehood (4–8 weeks) to adulthood (over 8 weeks).

### 2.14 Morphological investigations

One kidney of lupus mouse was used to analyse the absolute number and percentage of CD90+αSMA+ mesangial cells by flow cytometry. The other one was fixed and then embedded in paraffin. Then, longitudinal paraffin sections of kidney tissues were stained with hematoxylin and eosin and LAIR1 antibody via immunohistochemical staining.^19^, ^21^ The number of LAIR1+ glomerulus in kidney area (mm^2^) was evaluated by microscopic examination (Leica Microsystems, Wetzlar, Germany). One hundred glomerulus were calculated in each group. The severities of proteinuria, leukocyturia, hematuria, glomerulonephritis, interstitial nephritis, and vessels infiltration were scored using a modified macroscopic scoring system in the range 0–3 (0 = normal; 1 = mild; 2 = moderate; 3 = severe).^20^

### 2.15 Flow cytometric analysis for IL‐35‐regulated signaling pathway, mesangial cells, and Tregs

The mesangial cells from different treatments were fixed and permeabilized to incubate with antibodies including phosphorylated p38 (p‐p38) MAPK (T180/Y182), p‐ERK1/2 (p‐ERK) (T202/Y204) (BD Biosciences), p‐JAK2 (Tyr1008) (Cell Signaling Technology, Danvers, MA, USA), p‐STAT1 (Ser727), p‐STAT3 (Tyr705) (BioLegend), and p‐STAT4 (Tyr693) (Thermo Fisher Scientific) at 4°C for 15 minutes. After washing, the mean fluorescence intensity (MFI) was measured by flow cytometry. The expression of IL‐12Rβ2 (BD Pharmingen) and gp130 (R&D Systems, Minneapolis, MN, USA) on mesangial cells were also determined by flow cytometry assay.^3^ While, the MFI of p‐STAT1 (Ser727), p‐STAT3 (Tyr705), and p‐STAT4 (Tyr693) in mesangial cells from PBS, IL‐35p, and IL‐35n treatments could determine the activity of IL‐35‐regulated singling pathway. The percentage and absolute number of CD4^+^CD25^+^FoxP3^+^ Tregs from lupus mice with different treatments were exanimated by flow cytometry.^2^, ^3^

### 2.16 Plasma cytokine concentrations of lupus mice

Plasmas from lupus mice with different treatments were harvested and stored at −80°C prior to be subjected to a multiplex immunoassay. Using a Milliplex MAP assay kit (Merck Millipore, Billerica, MA, USA), several cytokines (i.e., TGF‐β, IL‐10, IL‐1β, MIG, IL‐12, IFN‐γ, TNF‐α, IL‐17A and IL‐6) were measured through a Bio‐Plex 200 suspension array system (Bio‐Rad Laboratories, Hercules, CA, USA).

### 2.17 Statistical analysis

Statistical analysis of in vivo and in vitro data was described in the section of each assay. Results were expressed as mean ± standard deviation (SD) for normally distributed data. Mann‐Whitney U‐tests were used to test the continuous variables. Comparison of different groups was performed with Kruskal‐Wallis analysis of variance (ANOVA), followed by Dunn's post‐test for comparing the differences and calculating a probability (p) value for each pair of comparison. All hypotheses were two‐tailed, and p‐values < 0.05 were considered significant. Data analyses were performed using GraphPad Prism (version 8.0 for Windows; GraphPad Software, La Jolla, CA, USA).

## 3 RESULTS

### 3.1 Proteomic analysis of urine sample and association between urine Lair1 levels and clinical parameters

**FIGURE 1:**
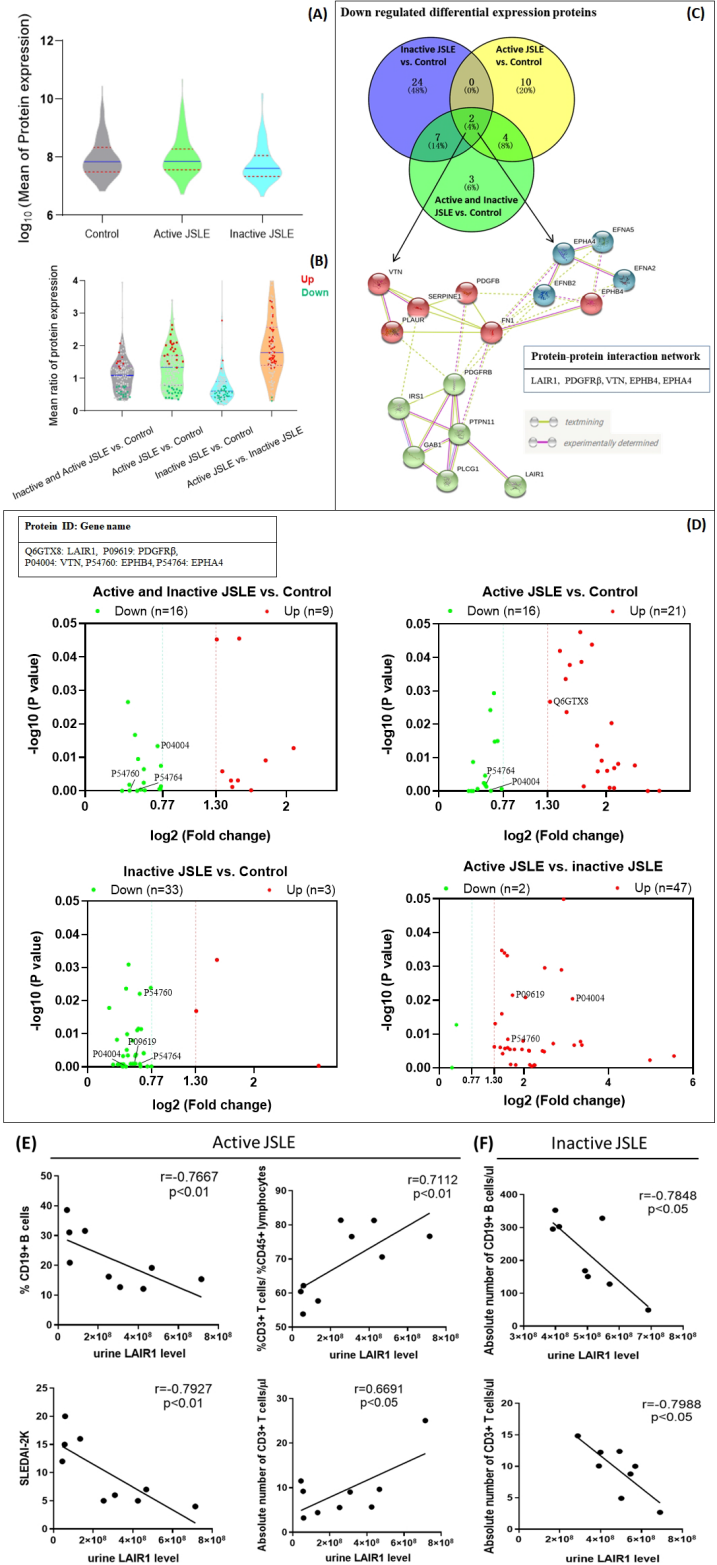
Differential expression profile of urinary proteins and the association between LAIR1 and clinical parameter. (A) Violin chart shows the mass spectrometric data of proteome as urinary differential expression proteins (DEPs) between JSLE and control groups. (B) Violin dots plot shows the ≥1.3 fold upregulated or ≤0.77 fold downregulated DEPs among active JSLE, inactive JSLE and control groups. (C) Venn diagram shows two downregulated DEPs between JSLE and control groups. The search tool for the retrieval of interacting genes (STRING) online database (https://string‐db.org/cgi/input.pl) tool shows this two DEPs involved protein‐protein interaction (PPI) network. (D) Dot plots show the significant upregulated and downregulated DEPs which involved in PPI network among groups. The −log10 (p value) plotted against the log2 (fold change) analysis thresholds are indicated by dotted lines (≥1.3 fold up‐regulation or ≤0.77 fold down‐regulation, p < 0.05). (E) Spearman's rank correlation test was used to assess the lineal associations among the urinary LAIR1 expression with %CD19+ B cells, %CD3+ T cells/%CD45+ lymphocytes, SLEDAI‐2K, and absolute number of CD3+ T cells in PBMC of active JSLE patients; as well as (F) with absolute number of CD3+ T and CD19+ B cells in PBMC of inactive JSLE patients. r = correlation coefficient

According to the bioinformatics analysis of proteome of collected urine sample, 105 DEPs among active JSLE‐LN patients (n = 10), inactive JSLE‐LN patients (n = 9) and healthy controls (n = 9) were found, in which several significantly up‐ (≥1.3 fold) and down‐ (≤0.77 fold) regulated interactive proteins like LAIR1, PDGFRβ, VTN, EPHB4, and EPHA4 were found through Venn and STRING analysis (Figures 1B–1D). Interestingly, upon the quantitative analysis of proteomic results (Figure 1E), we found that the urine LAIR1 levels were negatively correlated with %CD19+ B cells in PBMC (r = −0.6913, p < 0.05) and SLEDAI‐2K (r = ‐0.7927, p < 0.01) in active JSLE‐LN patients, as well as positively correlated with the ratio of %CD3+ T cells/%CD45+ lymphocytes (r = 0.7112, p < 0.01) and the absolute number of CD3+ T cells in the whole blood of active JSLE‐LN patients (r = 0.6691, p < 0.05). While, the similar negative associations were found between the urine LAIR1 level and the absolute number of CD19+ B cells (r = −0.7848, p < 0.05) and CD3+ T cells (r = −0.7988, p < 0.05) in the whole blood of inactive JSLE‐LN patients (Figures 1E–1F). Moreover, no significant associations were observed between the urine LAIR1 level and other index (data not shown). The detailed statistical differences of clinical characteristics between JSLE‐LN patients and healthy controls are demonstrated in Table 1 and Table S2.

**TABLE 1:** Demography of clinical characteristics for JSLE patients and healthy controls (mean ± SD)

Parameters	Healthy controls (n = 9)	Inactive JSLE (n = 9)	Active JSLE (n = 10)
Age	3.38 ± 1.02	9.80 ± 1.00*	10.80 ± 0.97*
Gender (M/F)	2/7	0/9	0/10
SLEDAI‐2K	NA	4.11 ± 4.08	11.50 ± 7.29^#^
%CD19+ B cells	NA	12.96 ± 5.61	21.41 ± 9.11^#^
Absolute number of CD19+ B cells/μL	NA	221.80 ± 111.40	181.40 ± 121.80
%CD3+ T cells/%CD45+ lymphocytes	NA	75.80 ± 6.27	69.96 ± 10.49
Absolute number of CD3+ T cells/μL	NA	1301 ± 466.90	569.10 ± 343.20^##^
%CD3^+^CD4^+^ Th cells	NA	29.60 ± 7.78	21.07 ± 8.40^#^
Absolute number of CD3^+^CD4^+^ Th cells/μL	NA	541.90 ± 261.80	190.00 ± 182.70^##^
%CD3^+^CD8^+^ cytotoxic T cells	NA	41.39 ± 4.93	46.26 ± 13.69
Absolute number of CD3^+^CD8^+^ cytotoxic T cells/μL	NA	693.40 ± 228.90	336.50 ± 154.10^##^
%CD16^+^CD56^+^ NK cells	NA	9.58 ± 6.91	7.89 ± 4.92
Absolute number of CD16^+^CD56^+^ NK cells/μL	NA	149.60 ± 100.10	61.24 ± 45.47^#^
Th/cytotoxic T cells of absolute number	NA	0.73 ± 0.22	0.52 ± 0.29
%CD4^–^CD8^–^ early T cells	NA	0.60 ± 0.23	1.05 ± 0.47^#^
Absolute number of CD4^–^CD8^–^ early T cells/μL	NA	9.48 ± 4.01	8.40 ± 6.73

The statistically significant differences were shown between JSLE patients versus healthy controls (^*^p < 0.05; ^**^p < 0.01), as well as the significant differences between the active and inactive JSLE patients (^#^p < 0.05; ^##^p < 0.01). There are no statistically significant differences in the parameters of gender, %CD3+ T cells/CD45+ Lymphocytes, CD3^+^CD8^+^ cytotoxic T cells, CD16^+^CD56^+^ NK cells, and the absolute number of CD19+ B cells, CD4^–^CD8^–^ early T cells, as well as Th/cytotoxic T cells of absolute number among groups.

### 3.2 Bioinformatics analysis for identifying the LAIR1 interactive proteins

**FIGURE 2:**
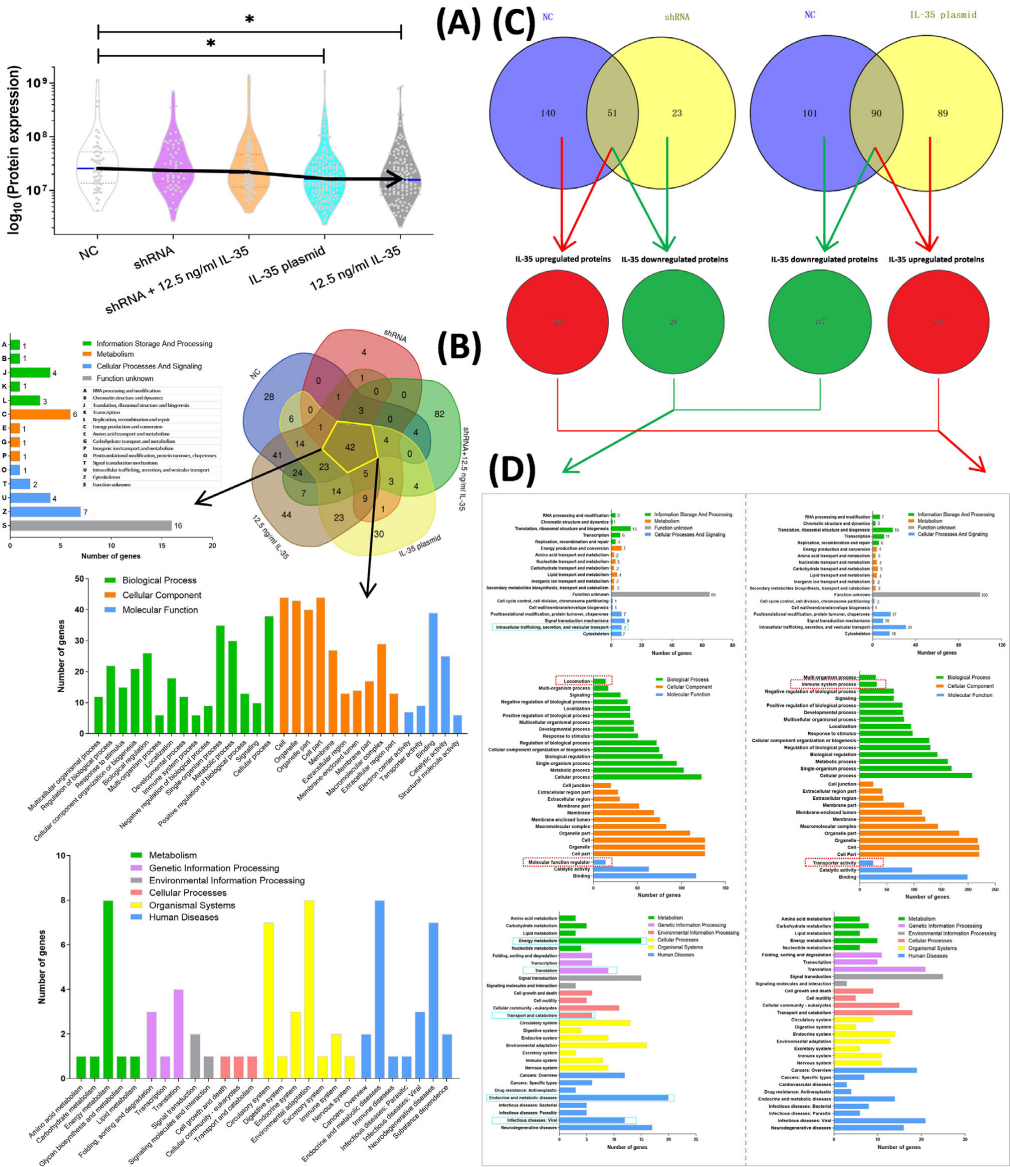
Identification of LAIR1 interactive proteins via Co‐IP with MS assay on mesangial cells with IL‐35 overexpression plasmid and shRNA treatments. (A) Violin dots plot shows expression of LAIR1 interactive proteins in different treatment groups by Co‐IP with MS assay. The black arrow line shows a trend of each group of log10 (protein expression). ^*^ p < 0.05, negative control (NC) versus other treatments. (B) Forty‐two common proteins among groups are analyzed by the annotation of clusters of orthologous groups (COG), gene ontology (GO) and Kyoto Encyclopedia of Genes and Genomes (KEGG). (C) Through Venn analysis, the ≥2 fold upregulated or ≤0.5 fold downregulated proteins are identified from the comparisons of shRNA versus NC and IL‐35 plasmid versus NC. The number of proteins is shown in the circle. (D) The COG, GO, and KEGG diagrams present the combined IL‐35 upregulated and downregulated proteins from Venn analysis. The cyan rectangles show the representative significantly different terms of COG and KEGG annotation. The red dash rectangles show terms difference between IL‐35 upregulated and downregulated proteins in GO annotation

When we analyzed the protein expression of mesangial cells with different treatments by Co‐IP assay, the decreased expressions of DEPs were found in IL‐35 plasmid and 12.5 ng/mL IL‐35 treatments (^*^p < 0.05, Figure 2A) rather than NC. Forty‐two common proteins among five different treatments were further analyzed for the annotation of COG, GO, and KEGG (Figure 2B). While, most of the common protein encoding genes in COG annotation were the function unknown genes. In addition, most of these genes were involved in energy metabolism, circulatory system, endocrine, and metabolic diseases in KEGG analysis (Figure 2B). Furthermore, when combined the IL‐35 up‐ and downregulated proteins from Venn analysis of shRNA versus (vs) NC and IL‐35 plasmid versus NC, we found 239 upregulated and 135 downregulated DEGs (Figure 2C). For further analysis of COG, GO, and KEGG annotation, there was a lower number of genes in IL‐35 downregulated DEGs than IL‐35 upregulated DEGs in COG annotation, such as intracellular trafficking, secretion, and vesicular transport (Figure 2D). Moreover, there were different numbers of IL‐35‐regulated DEGs which engaged the biological process including immune system process and locomotion, and the molecular function like transporter activity and molecular function regulator in GO annotation (Figure 2D). Similarly, when compared to IL‐35 upregulated DEGs in KEGG annotation, there was a lower number of DEGs in IL‐35 downregulated DEGs which involved in the translation, transport and catabolism, and viral infectious diseases, but a higher number of DEGs at energy metabolism, and endocrine and metabolic diseases (Figure 2D).

### 3.3 Interactive network between Lair1 and IL‐35‐regulated genes

**FIGURE 3:**
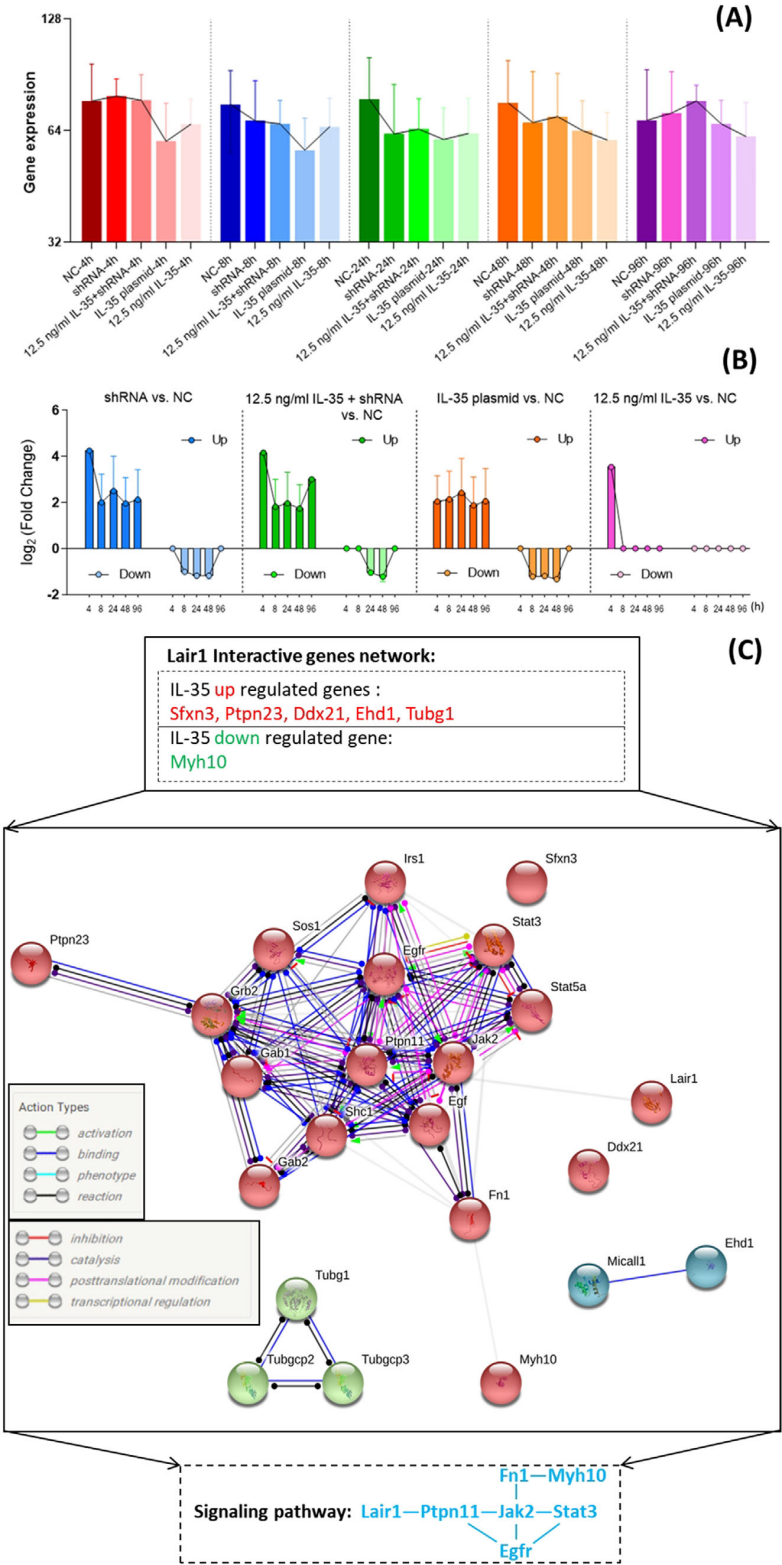
Presumed Lair1 interactive gene network. (A) Time‐series of gene expression among different treatments of mesangial cells is detected by RNA‐sequencing. These genes are derived from Co‐IP with MS identified LAIR1 interactive proteins in mesangial cells. (B) Time‐series of upregulated and downregulated gene expression between different treatment groups and NC. (C) A presumed signaling pathway of Lair1 interactive genes which are recognized from the above analysis of IL‐35 upregulated and downregulated genes by using the STRING online database

**FIGURE 4:**
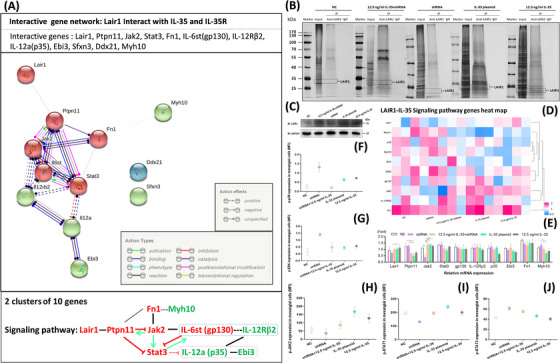
The interactive gene network of IL‐35, IL‐35R, and Lair1. (A) The components of IL‐35 and its receptor are involved in above presumed Lair1 interactive genes network. When exclude no interactive genes Ddx21 and Sfxn3, these 10 interactive genes can be classified into two clusters according to the STRING network analysis. The red and blue dash rectangles show the components of IL‐35 and its receptor, respectively. The red and green arrows represent inhibition and activation effects, respectively. (B) The immunoprecipitation analysis of LAIR1 and LAIR1 positive protein complex from NC, 12.5 ng/mL IL‐35+shRNA, shRNA, IL‐35 plasmid, and 12.5 ng/mL IL‐35‐treated mesangial cells. The bands in input line show total proteins lysed from mesangial cell with different treatments. IgG is the negative control for each IP. (C) The immunoblot assay for LAIR1 positive protein (31 kDa) in mesangial cell with different treatments. Glyceraldehyde‐3‐phosphate dehydrogenase (GAPDH) as loading control is 37 kDa. (D) Heat map shows the RNA‐seq results of 10 genes expression among different treatment groups. (E) The bar chart shows the RT‐qPCR results of the relative mRNA expression of 10 genes among different treatment groups (n = 3/group). (F and G) The mean fluorescence intensity (MFI) of phosphorylated ERK1/2 (p‐ERK) and p‐p38 MAPK in mesangial cells among different treatment groups are measured by flow cytometry. (H‐J) The MFI of p‐JAK2, p‐STAT1, and p‐STAT3 in mesangial cells among different treatment groups is presented as dot charts

According to the above findings of several Lair1 interactive genes, we have further investigated the IL‐35‐regulated DEGs in RNA‐seq by bioinformatics analysis. From the RNA‐seq results of mesangial cells with different treatments, there was an obvious downregulation of most DEGs in IL‐35 plasmid or IL‐35 (12.5 ng/mL) treatments at different time (Figure 3A). Meanwhile, a downregulation trend of IL‐35‐regulated DEGs was found from 4 to 48 hours in the comparison of NC and other treatments (Figure 3B). After combined these DEGs from Co‐IP and RNA‐seq to analyze in depth, we found five genes (Ddx21, Ehd1, Ptpn23, Sfxn3, and Tubg1) were upregulated by IL‐35, and only gene Myh10 was downregulated. However, from the analysis of interactive gene network between Lair1 and IL‐35‐regulated six DEGs (Ddx21, Ehd1, Ptpn23, Sfxn3, Tubg1, and Myh10) by using the STRING online database (Figure 3C), we found Myh10 and Ptpn23 could form an interactive gene network with Lair1 through several genes. However, the others (Ddx21, Ehd1, Sfxn3, and Tubg1) had no confident evidence to interact with Lair1 upon the analysis of STRING online database (Figure 3C). Therefore, a presumed signaling pathway of interactive gene network between Lair1 and IL‐35‐regulated DEGs (Myh10 and Ptpn23) could be established (Figure 3C). Furthermore, when adding the subunits of IL‐35 (Ebi3‐p35) and IL‐35R (gp130‐IL‐12Rβ2) to above presumed interactive gene network for further analysis, finally, we could propose a novel signalling pathway including 10 genes such as IL‐35, IL‐35R, IL‐35‐regulated genes and Lair1 (Figure 4A). Further analysis of Co‐IP results showed an obvious increase of total IP proteins (Figure 4B) and total LAIR1 (Figure 4C) in IL‐35 plasmid and 12.5 ng/mL IL‐35‐treated mesangial cells than NC and shRNA groups. When compared with NC and shRNA treatments, a similar downregulation of Ptpn11 and Myh10 was found in the IL‐35 plasmid‐treated mesangial cell by RNA‐seq and RT‐qPCR detection (*p < 0.05, Figures 4D and 4E). Furthermore, the results of upregulated p‐JAK2 (Tyr1008), p‐STAT1 (Ser727), and downregulated p‐STAT3 (Tyr705) in IL‐35 plasmid and 12.5 ng/mL IL‐35‐treated mesangial cells were verified by flow cytometry assay (Figures 4H–4J). This implied a confident evidence for the regulation of JAK/STAT signaling pathway by IL‐35 in mesangial cells. It was also proved that our assumed interactive network of Lair1, IL‐35, and IL‐35R was involved in this signaling pathway. Besides, the p‐p38 MAPK and p‐ERK were upregulated in shRNA‐treated mesangial cells and downregulated in IL‐35 plasmid treatment (Figures 4F and 4G), which may represent an inhibitory effect of IL‐35 on MAPK signaling pathway in mesangial cells.

### 3.4 Regulatory effect of IL‐35 and Tregs in lupus mice

**FIGURE 5:**
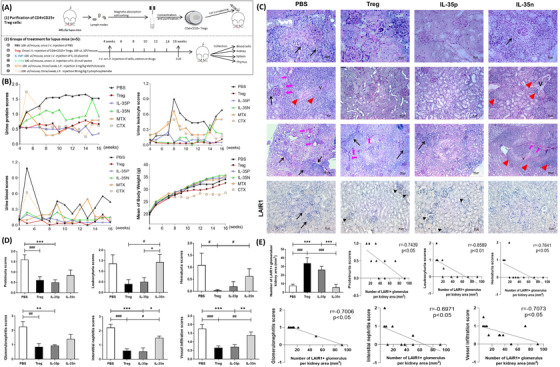
Regulatory effect of IL‐35 and Tregs in vivo. (A) The study design of IL‐35 and Tregs treatments in lupus mice model. (B) The characteristic parameters of lupus nephritis upon IL‐35 plasmid, Tregs and medicine treatments. (C) The representative histological staining of lupus mice kidney in different treatments including PBS, Treg, IL‐35p, and IL‐35n. Scale bars are 25, 50, and100 μm among groups, respectively. The black arrows denote the inflammatory cell infiltration around glomerulus. The pink arrows denote the protein effusion. The red triangles denote the vessels (V) infiltration. The black triangles denote the positive staining of LAIR1 in glomerulus. (D) The index scoring system of lupus nephritis, including proteinuria, leukocyturia, hematuria, glomerulonephritis, interstitial nephritis and vessels infiltration is presented as bar charts among different treatments. ^*^p < 0.05, ^**^p < 0.01, ^***^p < 0.001, IL‐35 overexpression plasmid (IL‐35p) versus other treatments; ^#^p < 0.05, ^##^p < 0.01, ^###^p < 0.001, Treg cells‐treated lupus mice versus others (n = 5 in each group). (E) The bar chart shows the number of LAIR1+ glomerulus/kidney area (mm^2^) in each group. ^***^p < 0.001, IL‐35p versus other treatments; ^###^p < 0.001, Treg cells‐treated lupus mice versus others (n = 5 in each group). Spearman's rank correlation test was used to assess the lineal associations among the number of LAIR1+ glomerulus per kidney area (mm^2^) in IL‐35p treatment with index scores of lupus nephritis, including proteinuria, leukocyturia, hematuria, glomerulonephritis, interstitial nephritis, and vessels infiltration. r = correlation coefficient

Since the Lair1 and IL‐35 involved interactive gene network and the regulatory effect of IL‐35 on JAK/STAT and MAPK signalling pathway were proved, the regulatory effect of IL‐35 and Tregs in vivo should be further investigated. According to the study design of IL‐35 and Tregs treatments in lupus mice (Figure 5A), the urine protein, leukocyte and blood scores in IL‐35p and Treg groups were kept at a very low level during the whole treated period of mouse life‐span from juvenilehood (4‐8 weeks) to adulthood (>8 weeks), when compared to medicine (MTX and CTX), IL‐35n, and PBS groups (Figure 5B). Meanwhile, although in the PBS group, these scores were gradually increased during the progression of LN, there were no obvious increments at the very beginning of juvenilehood (4‐8 weeks) in Tregs and IL‐35p‐treated mice, which implicated an overt therapeutic effect of Tregs and IL‐35p on LN. However, there may be a side effect of long‐term CTX treatment during adulthood (>8 weeks), due to an overt decrease of mice body weight, but no obvious side effect was found in other treated mice (Figure 5B). Furthermore, we found a significant decreased scores of proteinuria, leukocyturia, and hematuria, as well as a decrement of renal histopathological scores including glomerulonephritis, interstitial nephritis, and vessels infiltration (Figures 5C and 5D), in contrast, a significant increased number of LAIR1+ mesangial cells in IL‐35p (^***^p < 0.001) and Tregs (^###^p < 0.001)‐treated lupus mice rather than IL‐35n and PBS treatments (Figures 5C and 5E). Interestingly, when analyzed the lineal association between the number of LAIR1+ glomerulus/kidney area (mm^2^) and renal histopathological scores in IL‐35p‐treated lupus mice (Figure 5E), there was an obvious negative association among the number of LAIR1+ glomerulus/kidney area and scores of proteinuria (r = −0.7439, p < 0.05), leukocyturia (r = −0.8589, p < 0.01), hematuria (r = −0.7641, p < 0.05), glomerulonephritis (r = −0.7006, p < 0.05), interstitial nephritis (r = −0.6971, p < 0.05), and vessels infiltration (r = −0.7073, p < 0.05).

**FIGURE 6:**
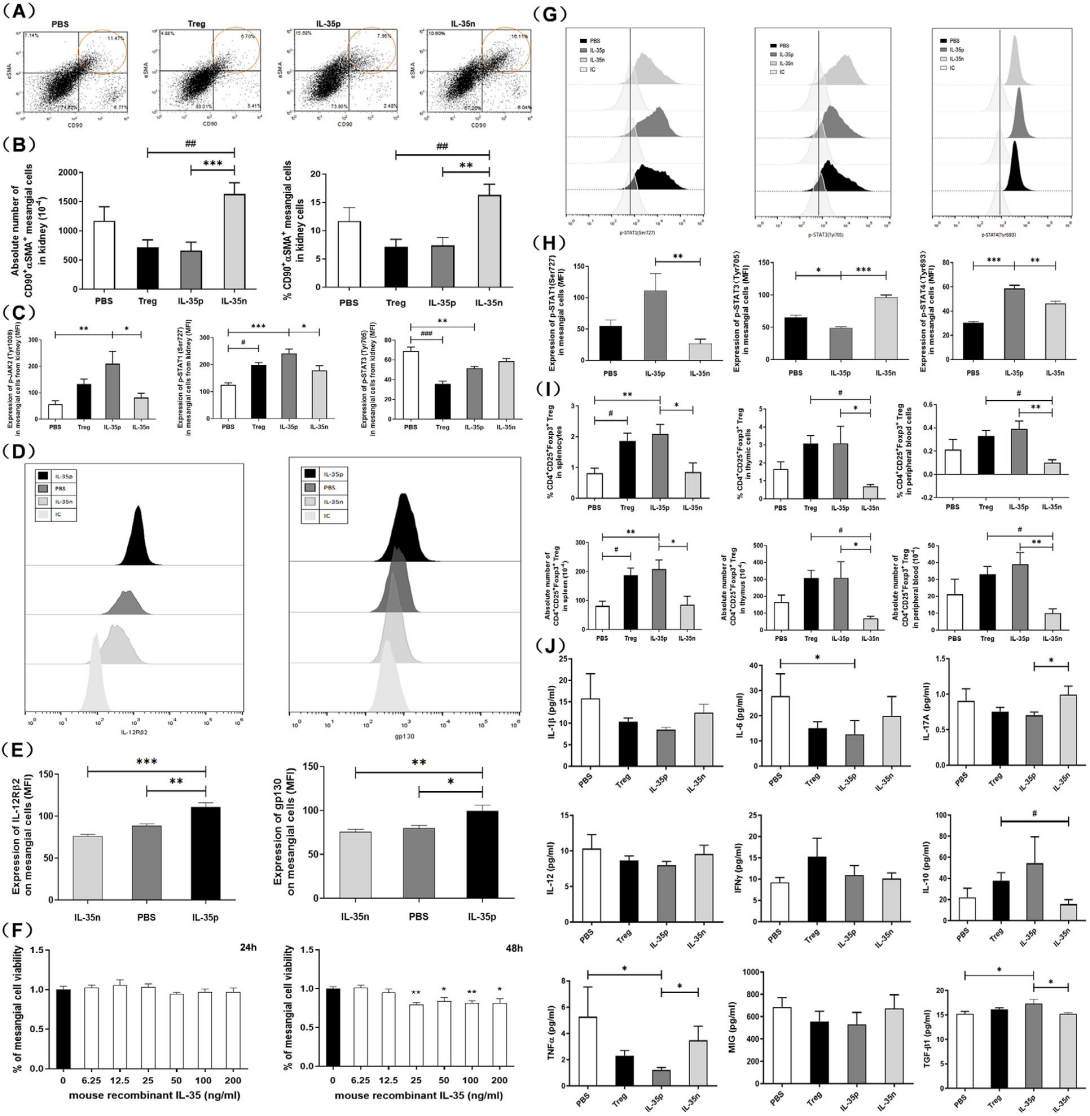
Regulation of JAK/STAT signaling pathway and inflammatory cytokines by IL‐35. (A) The representative dot plots are shown for the CD90^+^αSMA^+^ mesangial cells gated from kidney cells in different treatments. (B) The bar charts show the absolute number and percentage of CD90^+^αSMA^+^ mesangial cells among different treatments. ^**^ p < 0.01, ^***^p < 0.001, IL‐35p versus other treatments; ^##^ p < 0.01, Treg cells‐treated lupus mice versus others (n = 5 in each group). (C) The bar charts show the expressions of p‐JAK2 (Tyr1008), p‐STAT1 (Ser727), and p‐STAT3 (Tyr705) in mesangial cells among PBS, Treg, IL‐35p, and IL‐35n‐treated lupus mice (n = 5 in each group). ^*^p < 0.05, ^**^p < 0.01, ^***^p < 0.001, IL‐35p versus other treatments. ^#^ p < 0.05, ^##^p < 0.01, Treg versus other treatments. (D) The representative histograms of IL‐12Rβ2 and gp130 among IL‐35p, IL‐35n and PBS‐treated mesangial cells. IC is the isotype control. (E) The bar charts show the expressions of IL‐12Rβ2 and gp130 on mesangial cells among the above different treatments (n = 5 in each group). ^*^p < 0.05, ^**^p < 0.01, ^***^p < 0.001, IL‐35p versus other treatments. (F) The bar charts represent the percentage of mesangial cells viability upon mouse recombinant IL‐35 treatments for 24 hours and 48 hours (n = 10 in each group). ^*^p < 0.05, ^**^p < 0.01, IL‐35 (0 ng/mL) versus other treatments. (G) The representative histograms of p‐STAT1 (Ser727), p‐STAT3 (Tyr705), and p‐STAT4 (Tyr693) among IL‐35p, IL‐35n, and PBS‐treated mesangial cells. IC is the isotype control. (H) The bar charts show the expressions of p‐STAT1 (Ser727), p‐STAT3 (Tyr705), and p‐STAT4 (Tyr693) in mesangial cells among the above different treatments (n = 5 in each group). ^*^p < 0.05, ^**^p < 0.01, ^***^p < 0.001, IL‐35p versus other treatments. (I) The bar charts show the absolute number and percentage of CD4^+^CD25^+^Foxp3^+^ Tregs in spleen, thymus and peripheral blood among different treatment groups. ^*^p < 0.05, ^**^p < 0.01, IL‐35p versus other treatments; ^#^p < 0.05, Treg cells treated lupus mice versus others (n = 5 in each group). (J) The bar charts show the plasma level of pro‐ and anti‐inflammatory cytokines among different treatment groups. ^*^p < 0.05, IL‐35p versus other treatments; ^#^p < 0.05, Treg cells treated lupus mice versus others (n = 5 in each group)

In addition, in consistent with the previous results, the absolute number and percentage of CD90^+^αSMA^+^ mesangial cells from kidney in IL‐35p and Tregs‐treated lupus mice were significantly reduced (Figures 6A and 6B). Similar to the expression of p‐STAT1 (Ser727)/p‐STAT3 (Tyr705) in vitro (Figures 6G and 6H), the upregulated p‐STAT1 (Ser727) (^*^p < 0.05, ^***^p < 0.001), and downregulated p‐STAT3 (Tyr705) (^**^p < 0.01) implied a regulatory effect of IL‐35 on JAK/STAT signaling pathway in vivo (Figure 6C). According to the expression of IL‐35R on mesangial cells (Figures 6D and 6E), we found a significant upregulation of IL‐12Rβ2 and gp130 in IL‐35p group rather than IL‐35n and PBS groups (^*^p < 0.05, ^**^p < 0.01, ^***^p < 0.001). Meanwhile, the cell viability was also significantly decreased in 25–200 ng/mL mouse recombinant IL‐35 directly treated mesangial cells for 48 hours. Moreover, as we known IL‐35 could be secreted by CD4^+^CD25^+^Foxp3^+^ Tregs. From the results of an increased absolute number and percentage of Tregs at spleen (^*^p < 0.05, ^**^p < 0.01), thymus (^*^p < 0.05), and peripheral blood (^**^p < 0.01) upon IL‐35p treatment, we have confirmed a positive feedback effect of IL‐35 on Tregs in vivo (Figure 6I). Therefore, this double enhanced effect of IL‐35 on Tregs and JAK/STAT signaling pathway could contribute to the significant decrement of plasma concentration of proinflammatory cytokines, such as TNF‐α (^*^p < 0.05), IL‐6 (^*^p < 0.05), and IL‐17A (^*^p < 0.05) as well as a significant increment of anti‐inflammatory cytokines like TGF‐β and IL‐10 (^*^p < 0.05) in Tregs and IL‐35p‐treated lupus mice (Figure 6J).

## 4 DISCUSSION

The molecules with interaction network involved in the pivotal biological processes and signaling pathways of different stage of JSLE‐LN are not well studied.^22^, ^23^ In order to explore whether distinct DEGs involved in multiple molecular functions, biological processes, and pathways can enrich the pivotal development of active JSLE‐LN, we have collected and diagnosed the noninvasive urine samples to discover the specific DEGs by using proteomics assay with LC‐MS/MS. There were 105 specific DEPs found in the comparison between JSLE‐LN patients and healthy controls by bioinformatics analysis. Fortunately, from these significant up‐ and downregulated DEPs, we found LAIR1, PDGFRβ, VTN, EPHB4, and EPHA4 could form a complex PPI network (Figure 1C). Similar to some previous findings,^5^, ^24^ in our study, the level of urine LAIR1 was significantly increased in active JSLE‐LN patients when compared to healthy controls (Figure 1D). The urine LAIR1 should be a soluble form and had a similar affinity to collagens and cell membrane LAIR1.^25^, ^26^ The inhibitory effect of LAIR1 can be impeded through soluble LAIR1 by two mechanisms: first, by reducing the amount of LAIR1 on cell surface; and second, by releasing the soluble LAIR1 as a receptor antagonist which can competitively bind to collagen.^6^, ^26^ Therefore, this demonstration could be an explanation that the upregulated urine LAIR1 may come from mesangial cell surface in active JSLE‐LN patients. It is also explained the significantly decreased number of LAIR1+ glomerulus/ kidney area in PBS‐treated lupus mice rather than IL‐35p and Treg treatments (Figures 5C and 5E). Additionally, when compared to the NC and shRNA‐treated mesangial cells, the mRNA expression of LAIR1 upon 12.5 ng/mL IL‐35 treatment was obviously elevated (Figure 4E). Most importantly, when IL‐35 increased the amount of LAIR1 on mesangial cell membrane (Figure 5E), it would result in an enhancement of LAIR1 inhibitory signals including JAK/STAT signaling pathway. Therefore, IL‐35 may interact with IL‐35R to transduce the phosphorylation signals by JAK2‐STAT1/4 signaling pathway, and then may enhance the inhibitory effect of LAIR1 to decrease the proliferation of mesangial cells, suppress the massive inflammation, and eventually inhibit progression of SLE‐LN.

In consistent with the previous finding which showed a remarkably upregulated gene expression of IL‐35R‐related Stat1 and Stat4 upon IL‐35 treatment in vivo,^3^ we have confirmed an upregulated expression of gp130 and IL‐12Rβ2 in IL‐35p transfected mesangial cells (Figures 6D–6E), and an regulatory effect of IL‐35 on intracellular JAK/STAT signaling pathway by increasing p‐JAK2 (Tyr1008), p‐STAT1 (Ser727), p‐STAT4 (Tyr693), and decreasing p‐STAT3 (Tyr705) in IL‐35‐treated mesangial cells in vivo (Figure 6C) and in vitro (Figures 6G and 6H). Besides, further bioinformatics analysis of Co‐IP and RNA‐seq results indicated that Lair1 could have an interactive network with Sfxn3, Ptpn23, Ddx21, Ehd1, Tubg1, and Myh10, which were regulated by IL‐35 in mesangial cells (Figure 3C). However, Ptpn23 and Myh10 had a direct interactive relationship with previously assumed signaling pathway of LAIR1‐PTPN11‐JAK‐STAT though using the STRING online database. Fortunately, the subunits of IL‐35 (Ebi3‐p35) and IL‐35R (gp130‐IL‐12Rβ2) could also involve in this signaling pathway to form a presumed interactive network of LAIR1‐PTPN11‐JAK‐STAT‐IL‐35R‐IL‐35 via STRING analysis (Figure 4A). Otherwise, a previous study demonstrated that the LAIR1 could recruit PTPN11 (SHP‐2) to mediate a negative regulatory signal of LAIR1 to inhibit cellular functions of NK cells, effector T cells, and B cells.^11^, ^27^ Meanwhile, the genetic or pharmacological inhibition of PTPN11 could prevent the activation of JAK2/STAT3 signaling pathway.^28^ In our study, upon the upregulated LAIR1 and downregulated PTPN11 (Figures 4D and 4E) and p‐STAT3 (Figure 4J) in IL‐35p‐treated mesangial cells in vitro, as well as the upregulated LAIR1 (Figures 5C and 5E) and downregulated p‐STAT3 (Figure 6C) in IL‐35‐treated lupus mice in vivo, we believe IL‐35‐upregulated LAIR1 may also transduce a negative signal by inhibiting PTPN11 to suppress STAT3 activation in mesangial cells. All of these findings might imply that LAIR1 should be involved in IL‐35‐regulated JAK/STAT signaling pathway in JSLE‐LN. However, whether IL‐35 can regulate LAIR1 directly, and whether LAIR1 is a novel direct target of IL‐35 needs further investigation.

Therefore, to further confirm this presumed interactive network, we have investigated the regulatory effect of IL‐35 on lupus mice model. Our previous report has demonstrated that Treg‐secreted IL‐35 could bind to its receptor, and then to activate downstream genes for Tregs differentiation,^3^ thereby forming a feedback loop to promote IL‐35 expression and its function. Consistent with previous findings that IL‐35 can upregulate the %Tregs in spleen, thymus, and peripheral blood, as well as Treg differential‐related genes in lupus mice,^3^ in this study, we also found the IL‐35p‐treated lupus mice have a higher absolute number and percentage of Tregs in spleen, thymus, and peripheral blood (Figure 6I). The increased Tregs could suppress the activity of the residual effector T cells in vitro^29^, ^30^ and may prevent kidney damage in chronic inflammation of SLE.^31^ However, the loss of IL‐35 results in a reduction of suppressive capacity of Tregs in vivo.^14^ These findings suggest a potential regulatory role of IL‐35 in lupus mice.

Together with the further analysis results of RNA‐sEquation (Figures 4D–4J) and flow cytometry (Figures 6G and 6H), it suggested a confidential proof that IL‐35 could activate JAK2‐STAT1/4 signalling pathway and suppress the phosphorylation of STAT3 and MAPK signaling pathway to regulate inflammatory cytokines production and secretion in mesangial cell. Moreover, similar to our previous finding of the significant decrease of plasma proinflammatory cytokines (IFN‐γ, IL‐6, and IL‐17A) in IL‐35‐treated lupus mice,^3^ we also found a significant decrease of proinflammatory cytokines (TNF‐α, IL‐6, and IL‐17A) and a significant increase of anti‐inflammatory cytokines (TGF‐β1 and IL‐10) in IL‐35p and Tregs treatments (Figure 6J). In addition, since the CD90^+^αSMA^+^ mesangial cells are an important source of proinflammatory cytokines like IL‐6 and TNF‐α in kidney,^32^, ^33^ the reduced mesangial cells in IL‐35p and Tregs‐treated lupus mice indicate an immunoregulatory functions of IL‐35 in vivo (Figures 6A, 6B and 6F). Combined with the increased positive staining of LAIR1 on mesangial cells of glomerulus in the IL‐35p and Tregs‐treated lupus mice (Figures 5C and 5E), and negative association among LAIR1+ glomerulus/kidney area with the parameters of LN (Figure 5E), The urine LAIR1 may become one of novel and potential diagnostic biomarkers for the development of JSLE‐LN. Furthermore, all the above results and the close relationship between IL‐35 and Tregs suggest that IL‐35‐regulated intracellular JAK/STAT signaling pathway can become a potential target for JSLE‐LN disease therapeutics in future. However, whether IL‐35 can directly upregulate the expression of LAIR1 to promote its inhibitory function in JSLE‐LN by suppressing the activation of JAK‐STAT3 signaling pathway, and the exact transcriptional factors involved in this regulation during the development of JSLE‐LN need further study.

In summary, this study has illustrated a number of meaningful DEPs in the urine of JSLE‐LN patients, which provides a new insight into the diagnosis and development of JSLE‐LN. Through together the bioinformatics analysis with the evidences in vivo and in vitro, we firstly assumed LAIR1 could be a novel potential target of IL‐35‐regulated JAK/STAT signaling pathway in JSLE‐LN. Therefore, discovering a novel mechanism by which IL‐35‐regulated JAK/STAT signaling pathway in lupus mice model may contribute to the development of new clinical therapeutics for JSLE‐LN disease in future.

## CONFLICT OF INTEREST

The authors declare that there is no conflict of interest that could be perceived as prejudicing the impartiality of the research reported.

## ETHICS APPROVAL AND CONSENT TO PARTICIPATE

The human study was approved by the Ethics Committee of Guangzhou Women and Children's Medical Center. Written informed consent was obtained from all subjects according to the Declaration of Helsinki. All experiments involving live animals were carried out strictly according to the principles outlined in the Animal Experimentation Ethics Committee Guide for the Care and Use of Laboratory Animals, as approved by the Animal Experimentation Ethics Committee of CUHK.

## AUTHOR CONTRIBUTIONS

All authors made substantial contributions to study design, analysis and interpretation of data, drafting the manuscript, and editing for important intellectual content of this article. Study conception and design: Zhe Cai. Performed the experiments: Zhe Cai, Song Zhang, and Ping Wu. Acquisition of data: Song Zhang and Zhe Cai. Contributed reagents/materials/analysis tools: Ming Hong, Qi Ren, Ping Wei, Yu Feng, Zhe Cai, Chun Kwok Wong, Hong Tang, and Huasong Zeng. Wrote the paper: Zhe Cai and Chun Kwok Wong.

## Supporting information

SUPPORTING INFORMATION

SUPPORTING INFORMATION

## Data Availability

The datasets used and/or analyzed during the current study are available from the corresponding author on reasonable request.

## References

[1] Sui W , Hou X , Che W , Yang M , Dai Y . The applied basic research of systemic lupus erythematosus based on the biological omics. Genes Immun. 2013;14:133–146. 23446742 10.1038/gene.2013.3

[2] Cai Z , Wong CK , Kam NW , et al. Aberrant expression of regulatory cytokine IL‐35 in patients with systemic lupus erythematosus. Lupus. 2015;24:1257–1266. 25966926 10.1177/0961203315585815

[3] Cai Z , Wong CK , Dong J , et al. Remission of systemic lupus erythematosus disease activity with regulatory cytokine IL‐35 in MRL/lpr mice. Clin Exp Immunol. 2015;181:253–266. 25845911 10.1111/cei.12639 PMC4516441

[4] Arriens C , Wren JD , Munroe ME , Mohan C . Systemic lupus erythematosus biomarkers: the challenging quest. Rheumatology (Oxford). 2017;56:S32–S45. 10.1093/rheumatology/kew407 PMC5850341 28013203

[5] Bonaccorsi I , Cantoni C , Carrega P , et al. The immune inhibitory receptor LAIR‐1 is highly expressed by plasmatocytoid dendritic cells and acts complementary with NKp44 to control IFNa production. PLoS One. 2010;5:e15080. 21151495 10.1371/journal.pone.0015080 PMC2994815

[6] Kanakoudi‐Tsakalidou F , Farmaki E , Tzimouli V , et al. Simultaneous changes in serum HMGB1 and IFN‐α levels and in LAIR‐1 expression on plasmatoid dendritic cells of patients with juvenile SLE. New therapeutic options?. Lupus. 2014;23:305–312. 24399813 10.1177/0961203313519157

[7] Meyaard L . The inhibitory collagen receptor LAIR‐1 (CD305). J Leukoc Biol. 2008;83:799–803. 18063695 10.1189/jlb.0907609

[8] Meyaard L , Hurenkamp J , Clevers H , Lanier LL , Phillips JH . Leukocyteassociated Ig‐like receptor‐1 functions as an inhibitory receptor on cytotoxic T cells. J Immunol. 1999;162:5800–5804. 10229813

[9] Merlo A , Tenca C , Fais F , et al. Inhibitory receptors CD85j, LAIR‐1, and CD152 down‐regulate immunoglobulin and cytokine production by human B lymphocytes. Clin Diagn Lab Immunol. 2005;12:705–712. 15939744 10.1128/CDLI.12.6.705-712.2005 PMC1151979

[10] Wang JX , Mizui M , Zeng LF , Bronson R , Finnell M , Terhorst C . Inhibition of SHP2 ameliorates the pathogenesis of systemic lupus erythematosus. J Clin Invest. 2016;126:2077–2092. 27183387 10.1172/JCI87037 PMC4887187

[11] Meyaard L , Adema GJ , Chang C , et al. LAIR‐1, a novel inhibitory receptor expressed on human mononuclear leukocytes. Immunity. 1997;7:283–290. 9285412 10.1016/s1074-7613(00)80530-0

[12] Xu D , Qu CK . Protein tyrosine phosphatases in the JAK/STAT pathway. Front Biosci. 2008;13:4925–4932. 18508557 10.2741/3051 PMC2599796

[13] Chaturvedi V , Collison LW , Guy CS , Workman CJ , Vignali DA . Cutting edge: human regulatory T cells require IL‐35 to mediate suppression and infectious tolerance. J Immunol. 2011;186:6661–6666. 21576509 10.4049/jimmunol.1100315 PMC3110563

[14] Collison LW , Delgoffe GM , Guy CS , et al. The composition and signaling of the IL‐35 receptor are unconventional. Nat Immunol. 2012;13:290–299. 22306691 10.1038/ni.2227 PMC3529151

[15] Huang CH , Loo EX , Kuo IC , et al. Airway inflammation and IgE production induced by dust mite allergen‐specific memory/effector Th2 cell line can be effectively attenuated by IL‐35. J Immunol. 2011;187:462–471. 21613618 10.4049/jimmunol.1100259

[16] Reilly CM , Oates JC , Cook JA , Morrow JD , Halushka PV , Gilkeson GS . Inhibition of mesangial cell nitric oxide in MRL/lpr mice by prostaglandin J2 and proliferator activation receptor‐gamma agonists. J Immunol. 2000;164:1498–1504. 10640767 10.4049/jimmunol.164.3.1498

[17] Wilson HM , Stewart KN . Glomerular epithelial and mesangial cell culture and characterization. Methods Mol Biol. 2012;806:187–201. 22057453 10.1007/978-1-61779-367-7_13

[18] Lu YQ , Ye YT , Yang QQ , Shi SL . Single‐cell RNA‐sequence analysis of mouse glomerular mesangial cells uncovers mesangial cell essential genes. Kidney Int. 2017;92:504–513. 28320530 10.1016/j.kint.2017.01.016

[19] Feng Y , Cai Z , Cheung WK , et al. Multichromatic TTF staining characterizes cartilage matrix in osteoarthritisand bone development. Histol Histopathol. 2019;34:275–286. 30234227 10.14670/HH-18-044

[20] Chan OT , Hannum LG , Haberman AM , Madaio MP , Shlomchik MJ . A novel mouse with B cells but lacking serum antibody reveals an antibody‐independent role for B cells in murine lupus. J Exp Med. 1999;189:1639–1648. 10330443 10.1084/jem.189.10.1639 PMC2193634

[21] Cai Z , Feng Y , Li C , et al. Magnoflorine with hyaluronic acid gel promotes subchondral bone regeneration and attenuates cartilage degeneration in early osteoarthritis. Bone. 2018;116:266–278. 30149068 10.1016/j.bone.2018.08.015

[22] Aragon CC , Tafúr RA , Avellaneda AS , Martínez TMD , Salas ADL , Tobón GJ . Urinary biomarkers in lupus nephritis. J Transl Autoimmun. 2020;3:100042. 32743523 10.1016/j.jtauto.2020.100042 PMC7388339

[23] Stanley S , Vanarsa K , Soliman S , et al. Comprehensive aptamer‐based screening identifies a spectrum of urinary biomarkers of lupus nephritis across ethnicities. Nat Commun. 2020;11:2197. 32366845 10.1038/s41467-020-15986-3 PMC7198599

[24] Guo N , Zhang K , Cao XC , et al. Role and mechanism of LAIR‐1 in the development of autoimmune diseases, tumors, and malaria: a review. Curr Res Transl Med. 2020;68:119–124. 32690423 10.1016/j.retram.2020.05.003

[25] Lebbink RJ , van den Berg MC , de Ruiter T , et al. The soluble leukocyte‐associated Ig‐like receptor (LAIR)‐2 antagonizes the collagen/LAIR‐1 inhibitory immune interaction. J Immunol. 2008;180(3):1662–1669. 18209062 10.4049/jimmunol.180.3.1662

[26] Nordkamp MJMO , Roon JAGV , Douwes M , Ruiter TD , Urbanus RT , Meyaard L . Enhanced secretion of leukocyte‐associated immunoglobulin‐like receptor 2 (LAIR‐2) and soluble LAIR‐1 in rheumatoid arthritis. Arthritis Rheum. 2011;63(12):3749–3757. 22127695 10.1002/art.30612

[27] Lebbink RJ , De Ruiter T , Verbrugge A , Bril WS , Meyaard L . The mouse homologue of the leukocyte‐associated Ig‐like receptor‐1 is an inhibitory receptor that recruits Src homology region 2‐containing protein tyrosine phosphatase (SHP)‐2, but not SHP‐1. J Immunol. 2004;172:5535–5543. 15100296 10.4049/jimmunol.172.9.5535

[28] Zehender A , Huang JG , Györfi AH , et al. The tyrosine phosphatase SHP2 controls TGFβ‐induced STAT3 signaling to regulate fibroblast activation and fibrosis. Nat Commun. 2018;9:3259. 30108215 10.1038/s41467-018-05768-3 PMC6092362

[29] Tao R , Wang L , Murphy KM , Fraser CC , Hancock WW . Regulatory T cell expression of herpesvirus entry mediator suppresses the function of B and T lymphocyte attenuator‐positive effector T cells. J Immunol. 2008;180:6649–6655. 18453584 10.4049/jimmunol.180.10.6649

[30] Whibley N , Maccallum DM , Vickers MA , et al. Expansion of Foxp3(+) T‐cell populations by Candida albicans enhances both Th17‐cell responses and fungal dissemination after intravenous challenge. Eur J Immunol. 2014;44:1069–1083. 24435677 10.1002/eji.201343604 PMC3992851

[31] Wing K , Sakaguchi S . Regulatory T cells exert checks and balances on self tolerance and autoimmunity. Nat Immunol. 2010;11:7–13. 20016504 10.1038/ni.1818

[32] Su H , Lei CT , Zhang C . Interleukin‐6 signaling pathway and its role in kidney disease: an update. Front Immunol. 2017;8:405. 28484449 10.3389/fimmu.2017.00405 PMC5399081

[33] Ji M , Lu Y , Zhao C , et al. C5a induces the synthesis of IL‐6 and TNF‐alpha in rat glomerular mesangial cells through MAPK signaling pathways. PLoS One. 2016;11:e0161867. 27583546 10.1371/journal.pone.0161867 PMC5008626

