# Supplementary material for: A novel potential target of IL‐35‐regulated JAK/STAT signaling pathway in lupus nephritis
Source: Clin Transl Med. 2021 Feb 1;11(2):e309. doi: 10.1002/ctm2.309 (PMC7851357; doi:10.1002/ctm2.309)
Supplement: Supplementary file 1 — SUPPORTING INFORMATION [file CTM2-11-e309-s001.doc]

***Supplementary Table 1. A modified macroscopic scoring system of urinalysis for JSLE-LN***

| **Parameters** | P**roteinuria (mg/dl)** | **Leukocyturia (leukocyte/μl)** | **Urine blood (erythrocyte/μl)** |
| --- | --- | --- | --- |
| **Score 0** | 0-14 | 0-14 | 0-9 |
| **Score 1** | 15-29 | 15-69 | 10-24 |
| **Score 2** | 30-99 | 70-124 | 25-79 |
| **Score 3** | 100-299 | 125-499 | 80-199 |
| **Score 4** | 300-1999 | ≥ 500 | ≥ 200 |
