# Supplementary material for: A novel potential target of IL‐35‐regulated JAK/STAT signaling pathway in lupus nephritis
Source: Clin Transl Med. 2021 Feb 1;11(2):e309. doi: 10.1002/ctm2.309 (PMC7851357; doi:10.1002/ctm2.309)
Supplement: Supplementary file 2 — SUPPORTING INFORMATION [file CTM2-11-e309-s002.doc]

***Supplementary Table 2. Demography of clinical characteristics and medicine treatment of JSLE patients and healthy controls (mean±SD)***

| **Parameters** | **Healthy Controls (n=9)** | **Inactive JSLE (n=9)** | **Active JSLE (n=10)** |
| --- | --- | --- | --- |
| **Age** | 3.38±1.02 | 9.80±1.00* | 10.80±0.97* |
| **Gender (M/F)** | 2/7 | 0/9 | 0/10 |
| **SLEDAI-2K** | N/A. | 4.11±4.08 | 11.50±7.29^#^ |
| **Hematuria** | 0/9 | 1/9 | 5/10* |
| **Proteinuria** | 0/9 | 1/9 | 4/10 |
| **C3 (g/L)** | N/A. | 0.75±0.13 | 0.38±0.39^#^ |
| **C4 (g/L)** | N/A. | 0.14±0.04 | 0.06±0.05^##^ |
| **ANA (g/L)** | N/A. | 16.71±14.85 | 417.6±201.8^###^ |
| **dsDNA (g/L)** | N/A. | 14.52±12.27 | 443.1±309.7^##^ |
| **ESR (mm/h)** | N/A. | 29.11±22.86 | 6.50±6.76^#^ |
| **Creatinine (μmol/L)** | 22.87±4.37 | 61.13±15.99* | 33.30±1.90 |
| **Hemoglobin (g/L)** | 126.10±31.90 | 116.00±21.33 | 96.78±25.92* |
| **Cyclophosphamide (mg/d)** | N/A. | 180.0±44.72 | 208.3±20.41^#^ |
| **Hydroxychloroquine sulphate (mg/d)** | N/A. | 120.0±44.72 | 156.3±49.55 |
| **Prednisolone (mg/d)** | N/A. | 9.167±7.217 | 40.00±11.55^#^ |
| **Methylprednisolone (mg/d)** | N/A. | 8.400±12.12 | 20.00±16.20 |
| **Cyclosporin A (mg/d)** | N/A. | 32.25±36.39 | 0±0^#^ |
| **Mycophenolate mofetil (mg/d)** | N/A. | 750.0±273.9 | 625.0±250.0 |
| **Methotrexate (mg/d)** | N/A. | 6.667±2.887 | 8.750±1.443 |

The statistically significant differences were shown between JSLE patients versus healthy controls (*p<0.05), as well as the significant differences between the active and inactive JSLE patients (^#^p<0.05; ^##^p<0.01; ^###^p<0.001). There are no statistically significant differences in the parameters of gender, proteinuria and medicines like hydroxychloroquine sulphate, methylprednisolone, mycophenolate mofetil and methotrexate among groups. N/A, not available. C3/4, complement 3/4. ANA, antinuclear antibody. ERS, erythrocyte sedimentation rate.

|  |  |
| --- | --- |
|  |  |
